# Self-consistent field theory for the interactions between keratin intermediate filaments

**DOI:** 10.1186/2046-1682-6-12

**Published:** 2013-09-05

**Authors:** Anna Akinshina, Etienne Jambon-Puillet, Patrick B Warren, Massimo G Noro

**Affiliations:** 1 Unilever R&D Port Sunlight, Quarry Road East, Bebington, Wirral, CH63 3JW, UK; 2 School of Chemical Engineering & Analytical Science, The University of Manchester, Oxford Road, Manchester, M13 9PL, UK; 3 Institut Curie Centre de Recherche, CNRS UMR 168 - UPMC, F-75231, Paris Cedex 05, France

**Keywords:** Stratum corneum, Skin keratins, Intermediate filaments, Unstructured terminal domains, Bridging attraction

## Abstract

**Background:**

Keratins are important structural proteins found in skin, hair and nails. Keratin Intermediate Filaments are major components of corneocytes, nonviable horny cells of the Stratum Corneum, the outermost layer of skin. It is considered that interactions between unstructured domains of Keratin Intermediate Filaments are the key factor in maintaining the elasticity of the skin.

**Results:**

We have developed a model for the interactions between keratin intermediate filaments based on self-consistent field theory. The intermediate filaments are represented by charged surfaces, and the disordered terminal domains of the keratins are represented by charged heteropolymers grafted to these surfaces. We estimate the system is close to a charge compensation point where the heteropolymer grafting density is matched to the surface charge density. Using a protein model with amino acid resolution for the terminal domains, we find that the terminal chains can mediate a weak attraction between the keratin surfaces. The origin of the attraction is a combination of bridging and electrostatics. The attraction disappears when the system moves away from the charge compensation point, or when excess small ions and/or NMF-representing free amino acids are added.

**Conclusions:**

These results are in concordance with experimental observations, and support the idea that the interaction between keratin filaments, and ultimately in part the elastic properties of the keratin-containing tissue, is controlled by a combination of the physico-chemical properties of the disordered terminal domains and the composition of the medium in the inter-filament region.

## Background

The outermost layer of skin, the stratum corneum (SC), is often described as organised into a ‘bricks-and-mortar’ type structure, where the mortar represents the self-assembled lipid lamellae and the bricks refer to the protein-rich corneocytes [1-4]. Corneocytes are nonviable disk-shaped flat horny cells mainly composed of keratin proteins, organised in complex intermediate filament (IF) networks. Keratins, in turn, are important structural proteins which confer stiffness to many biological tissues such as skin, nails and hair. There are 54 functional human keratin genes, of which 28 are type I (acidic) keratin genes and 26 are type II (neutral and basic) keratin genes. A new systematic nomenclature and functional role of keratins was presented by Schweizer *et al.*[5], Moll *et al.*[6], and Gu and Coulombe [7].

**Figure 1:**
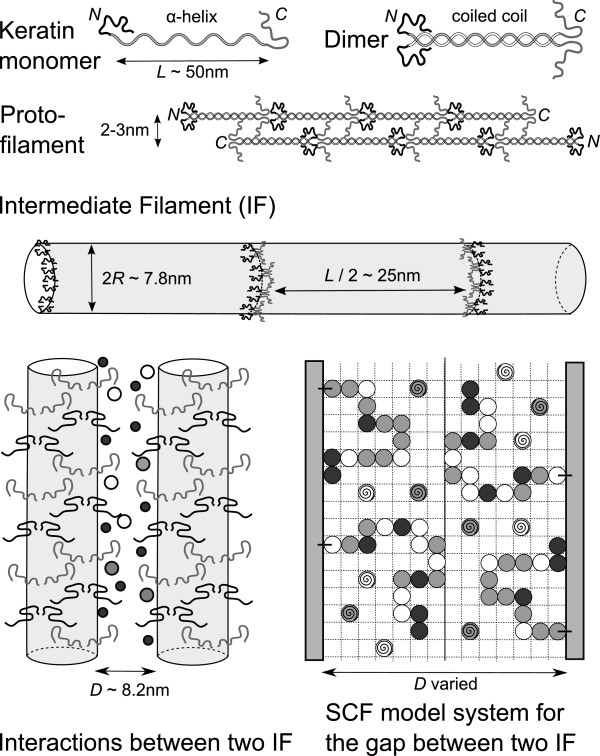
**IF organisation and the SCF model of N- and C-terminal domains attached onto IF surfaces.** The IF surfaces are modelled as plane walls, the grafted domains as connected monomers, the salt ions and/or free amino acids as single monomers. All the other space is occupied by water. The separation between walls is varied in order to obtain the interaction potential mediated by the walls with grafted domains.

Keratin monomers consist of central *α*-helical rod domains of similar substructure (≈310 amino acids) and two disordered (unstructured) glycine-rich N- and C-terminal domains of variable size. Two keratin polypeptides associate in a parallel arrangement to form an ≈50 nm long coiled coil dimer, consisting of two different types of keratins: one acidic (type I) and one neutral-basic (type II). The most frequent keratin (K) dimer expressed in the SC and the upper epidermis is the K1/K10 pair [8-10]. The two coiled-coil heterodimers further self-assemble into tetramers by packing into an antiparallel half-staggered configuration. Tetramers, in turn, aggregate end-to-end forming protofilaments with a diameter around 2– 3 nm. Two protofilaments make a protofibril with diameter of order 4– 5 nm; four of these assemble laterally to form the keratin IF with diameter of order 8– 10 nm [7,10-22]. Schematically, a keratin IF could be pictured as a long cylindrical object filled mainly by *α*-helical coiled coils domains, and decorated on the surface by disordered N- and C-terminal domains extending into the surrounding solution. An illustration of the IF hierarchical organisation is depicted in Figure 1.

Inside the corneocytes IFs are surrounded by a complex mixture of water, ions, free amino acids and other low molecular weight water soluble non-ionic compounds; this mixture is sometimes referred to as the “Natural Moisturising Factor” (NMF). NMF plays an important role in skin moisturisation and in maintaining the physico-chemical properties of the skin, such as elasticity and permeability [23-30]; and it results from proteolytic degradation of filaggrin, a histidine-rich protein [23-30]. A reduced amount of NMF correlates with dry, flaky and itchy skin. Dry skin conditions may be a cosmetic problem triggered by natural (seasonal) changes of SC physical properties [24,26], but they may escalate to severe inflammatory skin disorders such as atopic dermatitis [26,28], xerosis [26,29] ichthyosis [28,30] and psoriasis [28,30].

Jokura *et al.*[23] studied the effect of NMF on SC elasticity using NMR spectroscopy, rheology and electron microscopy. The authors observed that treating an excised SC sample with water releases NMF and leads to a keratin IF mobility reduction, and overall corneocyte rigidity. Electron micrograph evidence suggested that in the absence of NMF, keratin filaments tend to associated more tightly with each other. Further hydration of the sample does not improve the mobility of the fibers. However, the original IF mobility conditions were partially restored by application of amino acid solutions. The authors compared the effect of different types of amino acids on the restoration of the SC elastic properties: neutral or basic amino acids, such as glycine or lysine, provided remarkable recovery of SC elasticity. In contrast, acidic amino acid, such as aspartic acid, was not as effective.

These findings suggested the hypothesis that loss of SC elasticity is due to increased intermolecular attractive forces between keratin filaments. In physiological conditions, NMF plays the important role to reduce these attractive forces, and to ensure SC elasticity. It is tempting to argue the protruding non-helical regions (unstructured N- and C- domains) mediate the interaction between the NMF-rich matrix and the IFs. In this work we present a modelling study of the interactions between keratin IFs suspended in different media: (i) a salt free solution mimicking the NMF depleted system, and the effects of (ii) added salt and (iii) NMF-rich amino acid solution.

## Method

### Self-consistent field (SCF) approach

Interactions between two IFs formed by K1/K10 keratins were investigated using the lattice self-consistent field (SCF) method [31-37]. The helical cores of the two IF are modelled as planar surfaces at distance *D* apart with disordered N and C terminal domains uniformly grafted onto them. The space between the IF surfaces is filled by water molecules, ions and/or free amino acids. The schematic model system is illustrated in Figure 1.

In the lattice SCF scheme the space between two surfaces is divided into layers *z*=1,2,3,…,*D* parallel to the walls, and each layer is further divided into lattice cells of equal size. Each lattice site is occupied by one of the monomeric species of the system (i.e. by protein residue, water molecule, ion, etc.), so the total volume fraction for all the species in each layer equals one, $\sum_{\alpha }\phi^{\alpha }(z)=1$, where the volume fractions *ϕ*^*α*^(*z*) have the meaning of dimensionless concentration of species type *α* at the distance *z* from the surface. Obtaining the equilibrium concentration profiles for all the system components, *ϕ*^*α*^(*z*), is the primary target of the SCF calculations. The volume fraction distributions depend nonlinearly on the potential of mean force, *u*^*α*^(*z*), acting on each species *α* in the system. The potential for each component, *u*^*α*^(*z*), in turn, depends on the volume fraction profiles, as well as on the short range (Flory-Huggins) and long-range (electrostatic) interactions between all the species of the system. To find both quantities, *ϕ*^*α*^(*z*) and *u*^*α*^(*z*), a set of nonlinear equations is constructed and solved self consistently by an iterative procedure. The volume fraction profiles obtained in this way minimise the free energy of the system [35].

The SCF method is widely used to study properties of disordered proteins at interfaces. Earlier, the scheme was implemented to investigate adsorption of milk proteins, *β*-casein [38] and *α*_S1_-casein [39-42]. More recently the SCF approach was applied to protruding terminal domains of neurofilaments (NF) [43-47] and to microtubule-associated 3RS tau protein, expressed in neurons of the central nervous system [48]. The detailed description of the method can be found in the original literature. Here we apply the method to study unstructured terminal domains of skin keratin IF. We have considered and compared two models for terminal N and C domains, detailed next.

### Amino acid (AA) model for terminal domains

The first model (AA) is based on the primary structure of N and C terminal domains for the keratins K1 and K10. The amino acid sequence of these domains is taken from the Human IF Database [49]. The terminal domains of K1 (N1 and C1) consist of 180 and 151 amino acids, respectively, and of K10 (N10 and C10) of 146 and 124 amino acids. All the amino acids in this model are divided into five groups according to their properties: ‘H’, hydrophobic (Ala, Val, Leu, Ile, Met, Trp, Phe, Pro, Cys); ‘P’, polar (Ser, Thr, Tyr, Asn, Gln), ‘G’ (Gly); ‘ +’, basic (Arg, Lys, His); and ‘ −’, acidic (Glu, Asp). A similar approach for allocating amino acids into groups is widely used in literature [38-48].

As can be seen from the amino acid sequence, N and C domains for K1 and K10 are glycine-rich (∼50%), where glycine is mostly accumulated in blocks of 3–6 residues separated by one or two H or P residues. Being aware that glycine is a peculiar amino acid, showing both polar and hydrophobic behaviour (depending on the length of the poly-glycine residue) [50-53], we reserve for glycine a separate classification group ‘G’.

**Figure 2:**
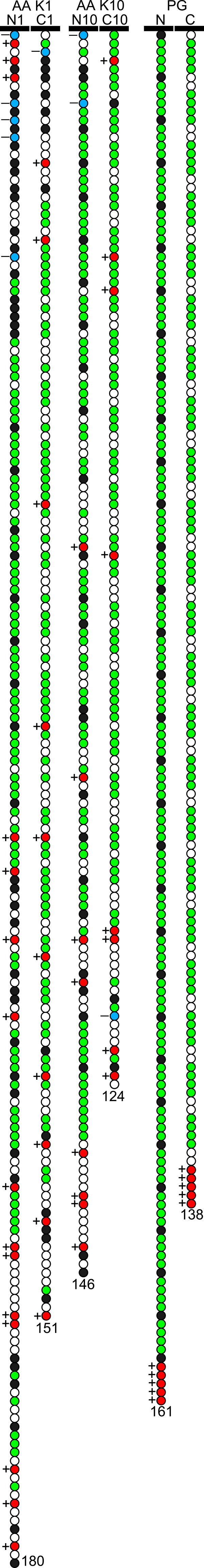
**Models of unstructured domains.** Schematic illustration of the N and C unstructured terminal domains for the AA and PG models. Colour code: white - “P”; black - “H”; red - “+”; blue - “–”, green - “G”.

The value of *p*H for SC is varied depending on SC depth, location and environment and the reported values of SC *p*H are in the order of 5–7 [54-56]. According to *p*K_*α*_ values, at *p*H=7 the amino acid residues Arg and Lys have charge *q*=+1 *e*, His has *q*=+0.36 *e*, and Glu and Asp have *q*=−1 *e*. At *p*H=5, Arg and Lys have charge of *q*=+1 *e*, His has *q*=+0.98 *e*, and Glu and Asp of *q*=−0.76 *e*. In view of the coarse grained level of the model, and to simplify the calculations, we consider that each basic residue carries the charge of *q*=+1 *e* and each acidic residue of *q*=−1 *e*. With such simplifications, the total charge of N1 domain is *q*_N1_=+10 *e* (+15 *e* and −5 *e*), for C1 it is *q*_C1_=+9 *e* (+10 *e* and −1 *e*), for N10 it is *q*_N10_=+6 *e* (+8 *e* and −2 *e*), and the charge of C10 is *q*_C10_=+7 *e* (+8 *e* and −1 *e*). The total charge of all four terminal domains will be *q*_NC_=+32 *e*. The AA model for all four domains is illustrated in Figure 2. In this study consider fixed (not *p*H-dependent) charges on the amino acids. We are aware that the electrostatic potential near the charged surface may affect the ionization of the acidic amino acids on the tails. However, most of our calculations are done under the conditions of low ionic strength where this effect is relatively small [48]. At higher ionic strength this effect may be more pronounced, but we believe that under such conditions the electrostatic screening effect would dominate.

### Polyglycine (PG) model for terminal domains

When examining the central parts of the residue sequence in the AA model in more detail, one can observe a repeating pattern of polyglycine blocks separated by one or several H, P or, rarely, basic monomers. One can also notice that the acidic residues are mostly located at the beginning of the tails (near the helical IF part, represented by planar surface in our SCF model) while the basic ones are mostly situated at the end of the tails (far from the IF surface). In order to capture and emphasize the major specific properties of the terminal domain structure we have designed a simplified “polyglycine” (PG) model for the N and C tails. The coarse PG model consists of repeating blocks of four G monomers and one H monomer (N tail) or four G and two P monomers (C tail) with additional five basic residues at the end of each tail. Thus, the structure of the N tail, N_PG_, is H_1_[ G_4_H_1_]_31_(+)_5_ and the structure of the C tail, C_PG_, is P_1_[ G_4_P_2_]_22_(+)_5_. The lengths of the N_PG_ and C_PG_ fragments are chosen to be 161 and 138 residues, respectively: this is because these numbers are near the average of N1 and N10 tail lengths (180 and 146 residues) for N_PG_ and, consequently, the average of C1 and C10 tail lengths (151 and 124 residues) for C_PG_. The N_PG_ and C_PG_ tail models are also illustrated in Figure 2.

### Modelling parameters

The short-ranged Flory-Huggins interaction parameters *χ* between the different types of monomers applied for both tail models are the follows. The hydrophobic residues, H, strongly repel all the polar ones, so we set *χ*=2 *k*_B_*T* for interactions of H with water and ions (Na, Cl), and *χ*=1 *k*_B_*T* for interactions of H with all the polar protein residues (P, +, −). The interactions of Na and Cl with water are attractive, *χ*=−1 *k*_B_*T* to mimic the tendency of hydration for the ions. Concerning the last residue group, G, we set *χ*=0.4 *k*_B_*T* for interactions of G and H group and *χ*=0.6 *k*_B_*T* for those between G and all the others residues (water, P, +, −, ions). The remaining interactions are set to be athermal (*χ*=0). All the monomer types considered have no affinity to the surface, *χ*_*s*_=0.

The choice of the interaction parameters for glycine is based on the experimental data for solubility of free glycine and glycine oligopeptides in water. Experimental evidence show that free glycine has rather good solubility in water [50-53,57]. However, the solubility of oligoglycines is much lower and it reduces with increase of the oligopeptide length [50,51,53]. Lu *et al.*[53] measured solubilities of glycine and its oligopeptides up to hexaglycine at different *p*H values and the results show that the solubility of oligoglycines longer than 3 residues strongly decreases with length. Bykov and Asher [51] reported that oligoglycines longer than 5 residues are normally insoluble in water; and Ohnishi *et al.*[50] stated that solubility of polypeptide with glycine linker beyond 6 is reduced and polyglycine segments longer than 9 residues form insoluble aggregates. In the current model, glycine is present in both forms: as oligomers in the sequence of keratin terminal domains and as free amino acid in the NMF composition. Taking into account dual hydrophobic-hydrophilic properties of glycine, we anticipate that the interactions of glycine with both hydrophobic and polar residues should neither be strongly repulsive nor attractive. Thus, we set the interactions with non-polar residues slightly attractive (*χ*=0.4 *k*_B_*T*) and with all polar slightly repulsive (*χ*=0.6 *k*_B_*T*). Alternatively, it would be possible to separate glycines into two groups: one for free glycine in NMF, and another one for glycine blocks in terminal domains, but that is beyond the scope of the current simplified model.

We have considered different values of dielectric permittivities, *ε*_*α*_, for different species components in our calculations. A similar approach has been used by Leermakers *et al.* for modelling projection domains of neurofilaments [43-47]. The permittivity for water was set to *ε*_*α*_=80, for hydrophobic group H and IF surface *ε*_*α*_=2, for all the polar and charged components (P, +, −, Na, Cl) *ε*_*α*_=5, and for glycine (G) we set *ε*_*α*_=4. The local dielectric permittivity was calculated according to $\varepsilon (z)=\varepsilon_{0}\sum_{\alpha }\varepsilon_{\alpha }\phi^{\alpha }(z)$, where *ε*_0_ is the permittivity of vacuum and *ϕ*^*α*^(*z*) is the volume fraction of species type *α* at distance *z*. The set of all parameters for both AA and PG models is given in Table 1.

**Table 1:** Flory-Huggins parameters

***χ***	**W**	**H**	**P**	**G**	***+***	***−***	**Na**	**Cl**	**s**	***q***	***ε***_***r***_
W	–									0	80
H	2.0	–								0	2
P	–	1.0	–							0	5
G	0.6	0.4	0.6	–						0	4
+	–	1.0	–	0.6	–					+1	5
−	–	1.0	–	0.6	–	–				−1	5
Na	-1.0	2.0	–	0.6	–	–	–			+1	5
Cl	-1.0	2.0	–	0.6	–	–	–	–		−1	5
s	–	–	–	–	–	–	–	–	–	(*)	2

Set of Flory-Huggins interaction parameters *χ* (in units of *k*_B_*T*), charges *q* (in units of *e*), and relative dielectric permittivities *ε*_*r*_. A dash ‘–’ indicates a zero entry. At (*) the surface (s) charge density (in units of *a*_0_^−2^) is varied between *σ*_s_=−0.0655*e* and *σ*_s_=−0.071*e* for the AA model (at *σ*_s_=−0.0664*e* the surface charge is fully balanced by the charge of the grafted chains), and between *σ*_s_=−0.0405*e* and *σ*_s_=−0.0425*e* for the PG model (at *σ*_s_=−0.0415*e* the surface charge is again fully balanced by the charge of the grafted chains).

The calculations were carried out using the lattice spacing of *a*_0_=0.4 nm. There are literature reports of lattice spacing ranging between values of 0.3 nm, used for modelling of caseins [38-42] and 0.6 nm applied for calculations of terminal domains of NF [43-47] and 3RS tau protein [48]. In our model system the main components are amino acids and water molecules. The water molecule size is about 3.1 Å and that of amino acids ranges from 3.9 Å for Gly to 6.1 Å for Trp [58]. As glycine, the smallest amino acid, is the main component of the sequence of the K1/K10 terminal domains, we used the intermediate value of *a*_0_=0.4 nm as lattice size in our calculations.

In this work we consider terminal domains uniformly grafted into two IF cores, which are represented by planar surfaces. The grafting density of the domains is calculated according to the fact that there are four terminal domains (two N and two C) per one dimer length of *L*=50 nm, and one IF core consists of 16 dimers (8 protofilaments), which gives in total 64 domains per dimer length. Taking the IF core diameter of 2*R*=7.8 nm [14] and the lattice size *a*_0_=0.4 nm, we obtain the grafting density $\sigma =64a_{0}^{2}/(2\mathrm{\pi LR})=0.0083$ (in units of $a_{0}^{2}$). For the AA model, with the average charge per each tail 〈*q*_N_〉=〈*q*_C_〉=+8 *e* (as the total charge of the four domains is *q*_NC_=+32 *e*), the charge density on the surface due to grafted chains would be *σ*_NC_=+0.0664 *e*. As for the PG model the charge density due to grafted chains would be, correspondingly, *σ*_NC_=+0.0415 *e*.

The calculation of the IF coiled-coil backbone charge is not so obvious due to lack of information about IF core organisation. Considering all the charged amino acids on the K1/K10 *α*-helical parts, we have obtained *N*_+_=45, *N*_−_=58 for K1 and *N*_+_=39, *N*_−_=60 for K10, which gives the net charge of the K1/K10 dimer *q*_dimer_=−34 *e*. We also take into account that 14 salt bridges do not change the total charge of the dimer. Carrying out the charge calculations for the IF core with dimer length of 50 nm, we consider, as previously, that IF backbone comprises 16 dimers in its cross section. Thus, we obtained the surface charge density (*i.e.* charge per $a_{0}^{2}$) of *σ*_s_=−0.071 *e*. This result for the surface charge density is appeared to be quite close to the value of the surface charge density due to the grafted chains, *σ*_NC_=+0.0664 *e* (with opposite sign). As explained by literature reports [12,13,59,60] the IFs are apolar; we expect that the overall charge of IF core, dangling terminal domains, and appropriate counterions should be balanced. Because the exact IFs organisation is unknown, we can not estimate how many of the accounted amino acids on the IF core are in their dissociated form. Keeping in mind that some parts of the protofilaments and, therefore, some of the charged amino acids could be hidden inside the IF core where is no water, we presume that the charge density of IF core could be lower then the calculated value of *σ*_s_=−0.071 *e*. Therefore, as a reference (starting) point for our calculations for the AA model we consider the surface charge density fully balanced with the charge density of the grafted chains, *σ*_s_=−0.0664 *e*. As for the PG model, the balanced value of the charge density would be *σ*_s_=−0.0415 *e*. We also explore a range of the surface charge densities around these values for both AA and PG terminal models. Salt is represented by added Na and Cl ions and the concentration was varied from as low as *c*_*s*_=10^−5^ M (*ϕ*_*s*_=3.8×10^−7^) to *c*_*s*_=0.1 M (*ϕ*_*s*_=3.8×10^−3^), depending on the system.

All the calculations were performed using the SCF code SFBOX kindly provided by Frans Leermakers.

## Results and discussion

### Overview

Structural organization of the N and C terminal domains in one surface and interactions between the two IF surfaces will be presented and discussed below. The results considered include volume fraction profiles, *ϕ*(*z*), of N and C terminals for both models, corresponding profiles of the basic residues, *ϕ*_+_(*z*), and free energy of interactions between the two IF surfaces with the attached terminal domains, *V*(*D*). We start with the case of balanced charge densities for the surface and chains, |*σ*_s_|=*σ*_NC_, at low ionic strength, *c*_*s*_=10^−5^ M. Then we consider the effect of added salt and discuss the options when the surface charge in absolute value is higher or lower than the charge on the grafted chains. At the end, in order to obtain better insights into the properties of each type of terminal domains, we consider the interactions of the surfaces with only one type of the chains (N or C) grafted.

### IF surfaces and tails at equal absolute charge: volume fraction profiles

**Figure 3:**
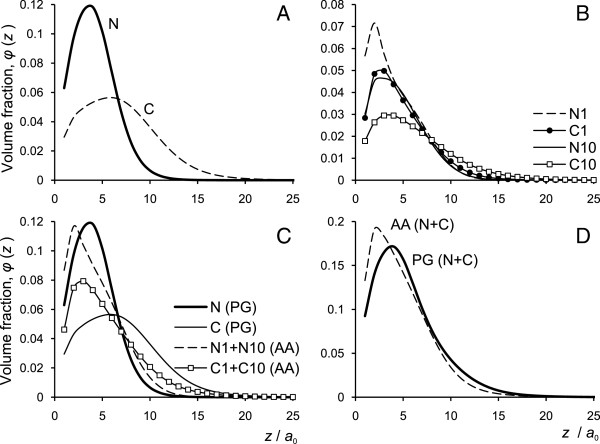
**Volume fraction profiles of the tails.** Volume fraction profiles for **(A)** PG model, **(B)** AA model, **(C)** comparison of volume fraction for both N tails (N1+N10) and both C tails (C1 and C10) for the AA model with N and C tails of the PG model, and **(D)** volume fraction of all the tails for the AA and PG models.

**Figure 4:**
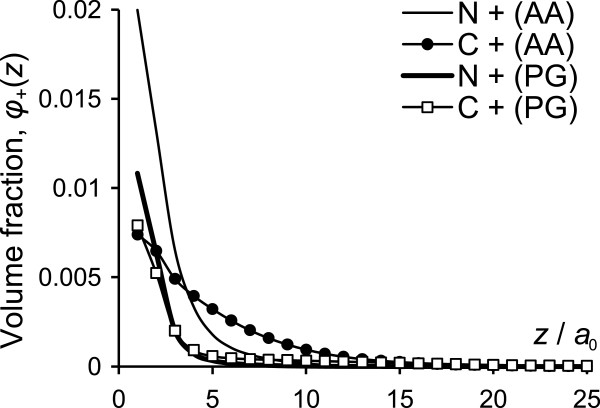
**Volume fraction of the basic residues.** Volume fraction of the basic residues on the N and C tails for the AA and PG models.

The volume fraction profiles, *ϕ*(*z*), show the monomer density of the grafted N and C domains at distance *z* from the surface. These distributions provide an estimate of how far the grafted chains extend from the surface and what is the most probable location of any specified monomers. The volume fraction profiles for the whole N and C chains are given in Figure 3 while Figure 4 shows the distributions for only positively charged monomers of N and C domains, *ϕ*_+_(*z*). In order to obtain the spatial distribution for an unaffected N and C chains, the profiles were obtained at large surfaces separation, so that the grafted chains do not interact (this corresponds to the limit of an isolated IF in solution).

#### Distribution of N and C tails

In Figure 3(A) we present the volume fraction profiles for the PG model of N and C tails. The profiles for the two tails are quite different: the monomer distribution of the more hydrophobic N tails is more narrow compared with the profile for C tails, with most of the monomers located in the first 10 layers from the surface and the maximum density at *z*=4 *a*_0_. The extension of the N tails does not exceed *z*=13 *a*_0_. More polar C tails have lower density near the surface and more extended profiles. The maximum density is slightly shifted away from the surface, *z*=6 *a*_0_, and the profiles extend up to *z*=20 *a*_0_. With the simple block-copolymer model for terminal domains we obtained the two distinct populations of the chains: (i) more hydrophobic N tails are collapsed near the surface and (ii) more polar C tails are projected farther into the solution. However, we should notice that both types of chains are actually quite compact near the surface. With contour lengths of 161 *a*_0_ and 138 *a*_0_ the chains do not spread out more than 13 *a*_0_ and 20 *a*_0_ respectively. We attribute this behaviour not only to the hydrophobic nature of both tails, major component of which is glycine, but also to the attraction of the positively charged end-monomers to the negatively charged surface, causing formation of loops.

The profiles for more realistic (AA) model for terminal domains, presented in Figure 3(B), show much smaller difference between the distributions for N and C tails. In general, the behaviour of the all four chains is similar: the distributions are quite narrow; most of the monomers are located within the first 10 layers from the surface, with the maximum density at *z*=2– 3 *a*_0_. The heights of the density maxima reflect chain lengths, with the highest maximum for the longest N1 tail and lowest one for the shortest C10. Having the contour length of 124– 180 *a*_0_ all the chains are in collapsed state and do not protrude far into the solution due to their hydrophobicity (∼50% of glycine) and the electrostatic attraction to the surface. The highest value of volume fraction is obtained for N1, the longest domain (180 *a*_0_). With the maximum in layer 2, the chains do not extend more than *z*=14 *a*_0_.

The high monomer density near the surface reflects the strong hydrophobic properties of N1 tail—with 24% of non-polar and 40% of glycine residues the chains prefer to be in compact conformation, reducing contacts with the polar solvent. Similar tail extension is observed also for N10 except that the maximum density value is lower than that for N1, because N10 chains are shorter and slightly less hydrophobic (17% of H monomers and 47% of G). The volume fraction profiles for C tails are slightly more extended than those for N tails. In particular, the distribution for C10 tail extends farthest, up to *z*=20 *a*_0_, and the monomer density near the surface is reduced. Even though C tails also consist of about 50% G residues, the fraction of non-polar H monomers is much smaller, 9% and 2% for C1 and C10, respectively. Being more polar than N tails, C tails extend a little farther into the solution.

In Figure 3(C) and (D) we compare the monomer distributions for the two models. Figure 3(C) shows the profiles separately for N and C tails and Figure 3(D) compares the total profiles for N+C tails together. The simplified PG model of the N tails gives the density distribution quite similar to the combined profile for N1+N10 tails, see Figure 3(C). Even though in the more detailed AA model the maximum is slightly closer to the surface and the extension of the profile is slightly larger (dashed line), these differences are comparatively small. As for the C tails, the difference between the profile for the PG model and the combined C1+C10 profile for the AA model is more pronounced. The general shape of the profiles is similar, so is their extension (to *z*=20 *a*_0_), but the density maximum for the PG model is lower and shifted away from the surface. That gives the impression that the hydrophilicity of the C tails in the PG model is somewhat overestimated; the more accurate AA model predicts that the C tails are more hydrophobic. Nevertheless, the relatively narrow profiles for the terminal domains coincide with the prediction of the compact structure of the tails due to formation of the glycine loops [61]. The glycine loops hypothesis predict that quasi-repetitive, glycine-rich terminal domains of epithelial keratins comprise flexible and compact glycine loops, where sequences of glycine make loops between the stacked non-polar residues. Even though SCF method does not allow obtaining such structural loops, it predicts compact conformation of the terminal domains near the surface. Therefore, despite some discrepancies in individual profiles for N and C tails, in the two models, the combined profiles for all (N+C) tails are fully consistent with each other, see Figure 3(D). The simple glycine multi-block model for N and C terminal domains reasonably well reflects the density distributions of terminal domains for K1/K10 IF.

#### Distribution of the basic residues of the tails

The volume fraction profiles for N and C domains provide the information about spatial distribution of the chains as a whole, while the location of the ends of the chains can be obtained from the distribution of the positive residues, *ϕ*_+_(*z*). The basic residues in the PG model are located only at the end of the tails and in the AA models they are scattered along the chains with higher concentration near the ends. The volume fraction profiles for basic residues in N and C tails, *ϕ*_+_(*z*), are presented in Figure 4.

For the PG model the distributions of positively charged monomers for N and C tails are practically the same. The positive monomers for both tails are located near the surface, with maximum at the first layer followed by abrupt decrease in the monomer density with the distance from the surface. For the distances *z*>5 *a*_0_ the fraction of basic monomers becomes very small. That result allows us to conclude that the basic residues, and, therefore, the end of the chains are located at the surfaces, so the tails form either loops back to the grafting surface or bridges with the opposite one.

As for the AA model, the distributions of positive residues for N and C tails differ both from those in the PG model and between each other. First, both distributions for the AA model are wider, especially for the C tails, and second, the difference between the *ϕ*_+_(*z*) profiles for N and C tails is more noticeable. For the N tails, *ϕ*_+_(*z*) is similar to that for the PG model, with the maximum at the first layer and subsequent decrease of the density with distance. At distances *z*>10 *a*_0_ very small fraction of basic monomers can be found. The total volume fraction is higher than that for the PG model because the amount of the positively charged monomers is higher. In the PG model there are only 5 basic monomers in each tail, while for N1 and N10 the numbers of basic monomers are 15 and 8, respectively. Taking into account that the grafting density of N tails for the PG model is the same as the sum of the grafting densities for N1 and N10, the calculated total amount of the positive charges for both N tails in the AA model is more than twice higher than that for the PG model. That results in about double the value of volume fraction of basic monomers for the AA model. Positively charged monomers for C tails distribute much wider and spreading gradually over ∼17 layers from the surface. The maximal density is again in the first layer but its value is more than half than that for the N tails, even though the number of positive charges for the C tails is not much smaller, 9 and 8 for C1 and C10, respectively.

The density profiles for all the tails show that the maximum density for the basic monomers is always at the first layer. The fact that the highest concentration of those residues is at the surface confirms our hypothesis that the charged monomers adsorb onto the surface, so the chains form loops and/or bridges between the surfaces. Broader volume fraction profiles of basic monomers for the AA model possibly result from the different distribution of the charged monomers along the chains. In the more detailed AA model, the basic monomers are not located exactly at the end of the chains, but somehow distributed along the whole length of the chains, with higher concentration at the ends. Thereby, the more uniformly distributed charges in the AA model give a thicker adsorbed layer while the clustered charges in the PG model adsorb flat on the surface, producing a very thin layer, similar to that of highly charged polyelectrolytes.

### IF surfaces and grafted tails at equal absolute charge: interaction potential profiles

The interactions between the two surfaces (IF cores) covered by grafted N and C terminal domains can be evaluated by calculating free energy of interactions between the surfaces at each separation *D*. The free energy of interactions *A*(*D*) is calculated from the partition function under conditions of restricted equilibrium, described by Evers *et al.*[37]. Under such conditions some components of the system are free to diffuse from the gap between the two surfaces to the bulk solution (e.g. water molecules, ions, free amino acids) and the others are restricted to stay within the gap (e.g. grafted N and C domains). The net interaction potential, *V*(*D*), is the difference between the free energy value at separation *D* and its value when the surfaces are far apart, *V*(*D*)=*A*(*D*)−*A*(*D*_*∞*_) and it is measured in units of $k_{B}T/a_{0}^{2}$. The “far apart” separation, *D*_*∞*_, is such that the two surfaces do not interact; in our calculations *D*_*∞*_ ranges between 150 *a*_0_ and 1000 *a*_0_, depending on the model and salt concentration. When the interaction potential is negative, *V*(*D*)<0, the two surfaces attract each other, while the positive potential, *V*(*D*)>0, implies the repulsive interactions between the surfaces. It can be shown that the interaction force between the two polymer-covered surfaces can be evaluated from the obtained interaction potential [37,62].

#### Low ionic strength

**Figure 5:**
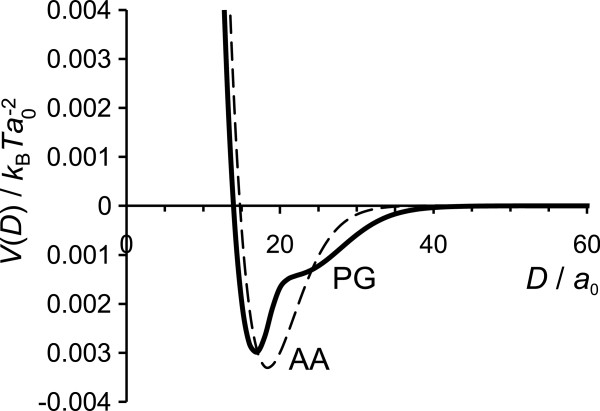
**Interaction potential in conditions of charge balance.** Interaction potential *V*(*D*) for the AA and PG models at the equal absolute charges of surfaces and grafted chains and low salt concentration, *c*_*s*_=10^−5^ M.

**Figure 6:**
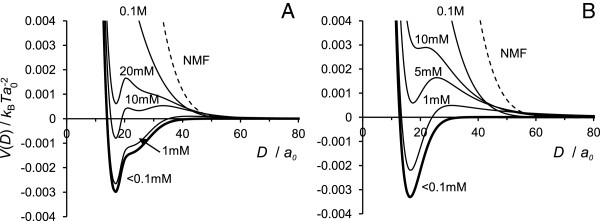
**Interaction potential for various ionic strength.** Interaction potential *V*(*D*) for **(A)** PG model, **(B)** AA model for various ionic strength indicated on the graphs at surface-chains charge balance conditions |*σ*_s_|=*σ*_NC_. Dashed lines represent data for NMF instead of salt.

The interaction potential for surfaces with attached N and C terminals (for both models) in conditions of charge balance between surface and chains, |*σ*_s_|=*σ*_NC_, is presented in Figures 5 and 6. There is no need for additional counterions to satisfy the charge neutrality condition. Ideally we would run the SCF calculation in the absence of added ions, but we are forced to introduce an extremely small concentration of added salt to maintain convergence. Still, the extremely low salt concentration case captures the experimental set up of two IF surfaces immersed in deionised water, where a small salt concentration cannot be avoided. Such is the case of the Jokura experiment [23] where the water extractable materials (NMF) from the SC sample were first released and then deionised water was added. Other extreme cases, where the surface charge is higher or lower than the charge on the terminal chains and addition of certain amount of salt (counterions) is required to obtain the charge neutrality, will be presented and discussed further below.

Figure 5 compares the interaction potential for both AA and PG models at low salt content, *c*_*s*_=10^−5^ M. For both models the interaction potential has a well pronounced minimum at *D*=17 *a*_0_ (6.8 nm) for the PG model and *D*=18 *a*_0_ (7.2 nm) for the AA model, corresponding to net attractive interactions in the system. It is interesting that the separations at which the attractive minimum occurs (*D*≈7 nm) are in agreement with the experimental values for the distance between the two IFs, *D*≈8.2 nm [14]. The attraction between the surfaces at separations *D*≈15– 35 *a*_0_ (where *V*(*D*)<0) occurs due to the well known polyelectrolyte bridging effect [34,37,63,64] and favorable electrostatic conditions (ionic strength). In the limit of low surface coverage, we believe that positively charged end-monomers are attracted to the opposite surface forming bridges across them. The possibility to be simultaneously attracted to more than one surface is more entropically favorable. The volume fraction profiles of the charged monomers discussed above support this picture as the positively charged residues are mostly located near the surface, which indicates the possibility of formation either loops or bridges (if the surfaces are close enough). At larger surface separations, *D*>35 *a*_0_ the interaction potential approaches zero, indicating that the grafted chains do not interact. However, at short separations, *D*<15 *a*_0_, the potential is positive due to strong steric repulsion between the chains.

We should draw attention to the fact that our simple PG model for N and C tails, based on the repetitive motif of glycine blocks, very well reproduces the result of the more complex AA model based on the amino acid sequence. Both characteristics of the system—the volume fraction profiles and the interaction potential between the IF surfaces—are in a good agreement between the two models. We believe that the PG model can be slightly improved, for example, by introducing some H residues into the C tail model and/or by distributing the charge less blockwise along the chain. Despite its simplicity, the PG model reflects well the properties of the N and C domains and, therefore, it probably can be used as a starting point for more refined (and computational intensive) modeling techniques, such as MC, MD or DPD.

#### High ionic strength and NMF

The interaction potential for the two models at different ionic strength is given in Figure 6. As we already discussed, at low salt content, *c*_*s*_<0.1 mM, the interaction potential develops an attractive minimum at short separations between the surfaces and levels to zero at longer separations. At higher ionic strength, *c*_*s*_≈1– 10 mM, the minimum becomes shallower and a repulsion apprears at larger separations. Our two models for terminal domains give qualitatively similar results but in the PG model more added salt is required to destroy the attraction, i.e. the PG model still shows a small attraction at *c*_*s*_=10 mM, while for the AA model the interactions are already repulsive at all separations at *c*_*s*_=5 mM. That occurs because the attraction to the surface of the charged block at the end of the PG chains is stronger than that of the AA chains (more uniform charge distribution along the chains), so more salt is needed to affect the attraction. In every case, at salt concentration near physiological conditions, *c*_*s*_=0.1 M, the strong repulsion between the surfaces with grafted chains is obtained for both models. High salt content leads to electrostatic screening, so the grafted chains mediate essentially a steric repulsion between the two opposing surfaces, much like polymer brushes. Obviously, at even higher ionic strength (*c*_*s*_>0.1 M) the repulsion between the surfaces becomes stronger and more short-ranged.

Calculations at varying levels of salt concentration were aimed at mimicking the Jokura experiments with normal (healthy) and reduced amounts of natural moisturizing factors (NMF) in SC. The amount of NMF is directly responsible for hydration level and elasticity of the skin [23-27]. NMF is made mostly of free amino acids derived from the enzymatic degradation of filaggrin, as well as organic and inorganic salts [23-30]. As charged amino acids and ions are important components of NMF, we have used high ionic strength as the first approximation. Increasing amounts of added salt tips the IFs interaction from attractive to repulsive. Experiments show that treatment with potassium lactate, could restore the SC hydration [24].

The next level of increasing complexity in our model was to account for the complex mixture of amino acids in the suspending matrix between the IFs. In order to create our coarse model of NMF we adopted the amino acid composition form Jacobson *et al.*[29] and then divided all amino acids into the same groups (H, P, G, +, −) used in the model for N and C tails. The water content was set to 30% as reported in the literature [23,27,65,66] and addition of neutraliser was necessary to ensure charge neutrality in the bulk. The detailed composition of this NMF + water model is given in Table 2.

**Table 2:** Composition of NMF solution

H	13.7%
P	25.5%
G	10.5%
+	8.4%
−	11.2%
(+)^∗^	2.8%
Water	27.9%
Total	100.0%

^∗^Neutraliser.

The interaction potentials for two IF surfaces with grafted terminals immersed into NMF solution are presented in Figure 6 by dashed lines. The graphs clearly illustrate that the more complex NMF-water mixture leads to an even stronger repulsion. We have observed that the mixture of free amino acids has stronger effect on the interactions between IF surfaces than just adding salt to the solvent; the NMF not only provides a strong repulsion between the approaching surfaces but also “pushes” the surfaces further away from each other. We believe the reason for such a strong repulsion between the surfaces rests in the high amount of free charged species (ions and amino acids), but what makes this forces more long ranged is the presence of free neutral amino acids. Solution of only neutral amino acids only slightly decreases the attraction between the surfaces and shifts of the attraction minimum to larger separations.

This result does not support the Jokura *et al.* finding that neutral amino acids improve mobility of keratin fibers, as well as basic amino acids, but not acidic ones [23]. Our results showed that only charged species in solution can affect the attractive intermolecular forces between negatively charged IF cores with grafted positively charged terminal chains. We should also mention, that in our coarse-grained model we could not reveal the specific effect of basic amino acids, as the properties of positive and negative free amino acids are the same except of the charge and the charge neutrality is required in the bulk. In order to examine the effect of specific ions a more sophisticated model and/or method is required.

### Surface charge higher or lower than the charge of the terminals

Previously we have described the case when charge on the surface is fully balanced by charge on the grafted chains only. As we already mention, some parts of the protofilaments and, therefore, some of the charged amino acids could be hidden inside the IF core and do not contribute to the charge density. That assumption may leads to the situation when the charges of the grafted tails and surface are not balanced. In order to account for such possibility, in this section we consider cases when the charge of the surface is higher or lower than the charge on the grafted tails. In order to obtain charge neutrality in under these conditions certain amount of counterions is needed. We do so by setting the concentration of salt *c*_*s*_ in the bulk, which is in equilibrium with the gap between the decorated IF surfaces.

**Figure 7:**
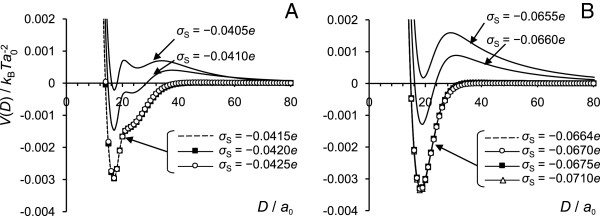
**Interaction potential for various surface charge.** Interaction potential for **(A)** PG model, **(B)** AA model for various surface charge indicated on the graphs neutralized by grafted tails and ions. The charge of the grafted chains is fixed via grafting density of *σ*=0.0083. The amount of salt needed for charge neutrality depends on the surface charge and is given in Table 3.

In Figure 7 we present the interaction potential, *V*(*D*), for the surface charge densities of *σ*_s_=−0.0655 *e*, −0.0660 *e*, −0.0664 *e*, −0.0670 *e*, −0.0675 *e*, and −0.0710 *e* for AA model and *σ*_s_=−0.0405 *e*, −0.0410 *e*, −0.0415 *e*, −0.0420 *e*, and −0.0425 *e* for the PG model. The charge on the grafted chains is kept constant at the values of *σ*_NC_=0.0664 *e* for the AA model and *σ*_NC_=0.0415 *e* for the PG model. Thus, for the AA model, surfaces with charge density |*σ*_s_|<0.0664 *e* are “undercharged” (in the specific sense that the surface charge density is smaller in absolute value than that needed to balance the charge on the grafted chains) and, correspondingly, with |*σ*_s_|>0.0664 *e* they are “overcharged”. For the PG model the threshold values of surface charge density for undercharged and overcharged surfaces would be, respectively, |*σ*_s_|<0.0415 *e* and |*σ*_s_|>0.0415 *e*.

**Table 3:** **Values for surface charge density (*σ***_**s**_**) and the corresponding salt concentration (*c***_***s***_**) required for charge neutralization**

	**PG**			**AA**	
*σ*_s_(*e*)	*c*_*s*_(M)	*Δσ*	*σ*_s_(*e*)	*c*_*s*_(M)	*Δσ*
−0.0405	3×10^−3^	0.0010	−0.0655	10^−3^	0.0010
−0.0410	10^−3^	0.0005	−0.0660	3×10^−4^	0.0005
−0.0415	10^−5^	0	−0.0664	10^−5^	0
−0.0420	5×10^−4^	−0.0005	−0.0670	5×10^−4^	−0.0006
−0.0425	10^−3^	−0.0010	−0.0675	10^−3^	−0.0011
			−0.0710	4×10^−3^	−0.0046

The difference between the charge densities of the surface and grafted tails is denoted by *Δσ*.

For each surface charge we found the values of the salt concentration which provide charge balance. The surface charge densities with the balancing salt concentrations are given in Table 3.

In the cases of under- or overcharged surface at low salt concentrations, repulsive electrostatic forces dominate, so the bridging attraction between the covered surfaces could not be seen. At high ionic strength, the repulsion decreases due to screening. When the surface is overcharged, i.e. when the charge on the surface is higher in absolute value than the charge of the chains, it is possible to find balancing salt concentration, under which the interaction potential between the surfaces would be the same as for the case when the surface charge is fully balanced by the charge of the chains only. The graphs in Figure 7 show that at certain amount of added salt the interaction potential profiles for |*σ*_s_|≥0.0664 *e* for the AA model and for |*σ*_s_|≥0.0415 *e* for the PG model completely overlap. Table 3 also shows that the stronger the charge imbalance (difference between surface and chains charge), the higher the amount of salt is required to neutralize the charge in the system. However, when the surface is undercharged, the attractive part is reduced and the potential always displays long-ranged repulsion, which increases with increasing charge imbalance. This phenomenology is the consequence of charge screening, as the following simplified model calculation shows. Consider a plane surface with a negative surface charge density *σ*_s_, surmounted by a charge cloud at charge density *σ*_NC_ uniformly distributed over a thickness *H*. We solve the linearised Poisson-Boltzmann equation for this problem,

(1) $$\frac{d^{2}\varphi }{dz^{2}}-\kappa_{s}^{2}\varphi =\left\{\begin{array}{ll} -4\pi l_{B}\sigma_{\text{NC}}/H & \;(0<z<H)\\ 0 & \;(z>H) \end{array}\right)$$

where *φ* is the electrostatic potential (in units of *k*_B_*T*/*e*), *κ*_*s*_ is the inverse Debye screening length ( $\kappa_{s}^{2}=8\pi l_{B}c_{s}$), and *l*_B_ is the Bjerrum length (*l*_B_≈0.72 nm). The boundary conditions are *dφ*/*dz*=−4*πl*_B_|*σ*_s_| at the wall and *φ*→0 as *z*→*∞*, and *φ* should be continuous at *z*=*H* with a continuous first derivative. This problem can be solved analytically. The behaviour of the potential at distances *z*>*H* from the surface is the relevant piece of information,

(2) $$\varphi =\frac{\sigma_{\text{NC}}\sinh(\kappa_{s}H)-|\sigma_{s}|\kappa_{s}H}{2{\text{Hc}}_{s}}\times e^{-\kappa_{s}z}\;(z>H)\;.$$

The prefactor indicates there is a special balance point where the potential vanishes completely for *z*>*H*. This point occurs when |*σ*_s_|/*σ*_NC_= sinh(*κ*_*s*_*H*)/(*κ*_*s*_*H*). The right hand side is an increasing function of *κ*_*s*_*H*, and only approaches unity for *κ*_*s*_*H*→0. Thus we see the surface has to be overcharged in order to reach the balance point and a higher degree of overcharging requires a larger value of *κ*_*s*_*H* to compensate, corresponding to higher salt, exactly as found above. The reason for this is that for *z*≥*H* the surface charge density is screened by an factor $∼e^{-\kappa_{s}H}$ relative to the diffuse oppositely-charged cloud.

The interactions between negatively charged surfaces covered by positively charged polyelectrolytes were investigated experimentally [67,68] by Monte-Carlo simulations [68], and theoretically [69]. The results of these studies have shown that the attractive bridging can dominate only when the charges of the polymers and ions balance the charge of the surface. Claesson and Ninham [67] demonstrated that attractive forces between mica surfaces covered by adsorbed chitosan were observed only when electrostatic double layer disappeared, i.e. when surface charges are exactly balanced by the charge of adsorbed polysaccharide. When charge of chitosan, controlled via variation of solution *p*H, was higher or lower than the charge of the mica surfaces, the electrostatic double layer repulsion forces dominate. Dahlgren *et al.*[68] measured the force acting between two mica surfaces covered by MAPTAC polyelectrolyte and also carried out MC simulations for two surfaces covered by oppositely charged polyelectrolytes. When PE adsorption was such that the surface charge was balanced by the polyelectrolyte, a strong attractive force was observed at short surface separations. Addition of salt to the MAPTAC solution facilitates the increased adsorption of polyelectrolyte, that leads to a reduced attraction and the appearance of a repulsive double-layer force. The authors concluded that the attractive bridging mechanism will only dominate when the polyelectrolyte adsorption approximately neutralizes the surface charge density. Borukhov *et al.*[69] proposed a theoretical approach to explain the behaviour of polyelectrolytes between charged surfaces. Their calculations show that at low ionic strength the attractive interactions between the surfaces take place when polymer adsorption balances surface charge. At high ionic strength the surface charge is balanced both by polymers and ions and the stronger the polymer charge, the more salt is needed to achieve the charge neutrality. The authors also considered values of adsorbed polymer higher or lower than the equilibrium adsorbed amount. When the adsorbed amount was lower than the equilibrium one, the attraction was weaker. However, when the adsorbed amount was higher than the equilibrium one, the results show stronger attraction between the walls and also appearance of strong long-ranged repulsion, similar to those shown in Figure 7.

In the experiments described by Jokura [23] loss of elasticity was observed for SC samples with extracted NMF and further hydrated by addition of deionised water. The authors suggested that loss of elasticity happens due to attractive intermolecular forces between keratin fibers. NMF, mainly free amino acids, reduces intermolecular forces through nonhelical regions of keratins (N and C terminal domains), so the keratin filaments acquire their elasticity. Our modelling results, theoretical consideration and literature analysis [67-69] show that the attractive interactions between IF at low salt content occur only when |*σ*_s_|=*σ*_NC_ or the IF surfaces are slightly overcharged. We conclude that the Jokura experiments could take place only at condition that surface charge is equal or slightly higher than the charge on the nonhelical chains. Thus, the charge of IF cores could not be much higher or lower than the charge on the unstructured terminal domains.

### Role of each type of terminal domains

Why has Nature used two types of unstructured terminal domains of similar length scale for each keratin protein? Does each domain type has a specific function and, if so, what is it? Is it necessary to capture the specific differences in a model? Different authors have taken different approaches. In modelling neurofilament projection domains [43-47], the much shorter globular N domains were not included in the study; only the C projection domains were considered. On the contrary, in 3RS tau protein research [48] the authors focused on the 196 amino acid long unstructured N domains. Thus, as the role of each domain in keratins is yet unknown, in order to generate insights, we decided to take advantage of fast computer models and examine the interactions mediated by each type of terminal domains separately.

**Figure 8:**
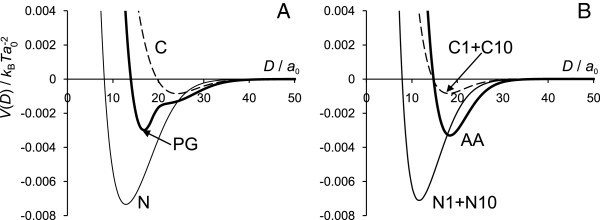
**Interaction potential for N and C terminal domains separately. (A)** for PG model, **(B)** for AA model. The curves for the cases when all the tails are present are also given for comparison (bold lines).

Figure 8 shows the interaction potentials for the IF cores grafted only with N domains or only C domains. The graphs from Figure 8 summarize the results and compare them against the full model calculations. For the calculation of only one type of domains, the grafting density of the chains was kept the same as before, *σ*=0.00415; this is half the total grafting density for both chain types together (*σ*=0.0083). The charge on the surface was then adjusted to neutralize the charge from the chains, *σ*_s_=−0.0332 *e* for the AA model and *σ*_s_=−0.02075 *e* for the PG model.

When only N chains are present, the minimum becomes much deeper and is shifted closer to the surface. Even with the addition of 0.1M of salt this attraction minimum is still quite deep (data not shown). Apparently, the more hydrophobic N tails behave as a “glue”, holding together the two surfaces. In contrast, the C tails behave in the opposite way. The interaction potentials for a similar model including C-tails only result in much smaller attraction minimum, pushed away from the surface. The more polar C-tails contribute much less to the attraction between the IF surfaces.

It is tempting to propose that both N and C domains play important roles in the structure and interactions of skin keratin IFs. The more hydrophobic N chains bring about a strong attraction between the IF surfaces while the more polar C tails push the surfaces away from each other, so that the two types of domains work together to keep IFs at the optimal separation. Therefore, we believe that it is the combination of both types of the domains balances the interactions between the intermediate filaments.

## Conclusions

We have applied the SCF approach to study interactions of the unstructured N and C terminal domains of skin keratin (K1/K10) Intermediate Filaments. Positively charged N and C domains were grafted onto negatively charged IF cores, represented by planar surfaces. We have considered two models for N and C tails, characterized by a different level of detail: the coarse block-copolymer PG model and the more detailed AA model, which is based on the amino acid sequence. In spite of the apparent simplicity of the PG model, it qualitatively captures most of the effects observed for the more complex AA model. We have presented monomer density profiles for the N and C tails and, separately, profiles for their basic residues only. We have compared and discussed interaction potential profiles for IF surfaces with attached tails at various surface charge densities, ionic strengths, and for the solution of free amino acids representing NMF. We have also attempted to clarify the role of each type of terminal domains considering N and C chains separately. Our main findings are summarized as follows. 

(A) Volume fraction profiles for N and C domains show that the monomers of both types of the chains are mostly concentrated near the surface, so the chain extension does not exceed *r*≈20 *a*_0_=8 nm (Figure 3). The basic residues of the terminal domains, which are located near the end of the chains, have the highest density at the (oppositely charged) surface (Figure 4). These results indicate that the chains form either loops or bridges with another surface. Such bridges lead to attractive interactions between the two IF surfaces at short separations. N tails are more hydrophobic and the profiles for N tails are more narrow compared to those for C tails and extend for no more than *r*≈15 *a*_0_ from the surface. The interaction potential for surfaces covered by N domain type only reveals that the attractive interactions between the surfaces are stronger than those when both types of the domains considered together and appear at shorter separations (Figure 8). So we conclude that N tails work as the “glue” between IF surfaces. C tails are slightly more polar than N tails and extend slightly further into the solution (*r*≈20 *a*_0_). The interactions between surfaces with only C tails grafted show much weaker and more long-ranged attraction. So, we propose that C chains are “responsible” for keeping a certain distance between IF. Hence, each type of the terminal domains has its specific role and their combination retain IF at certain distance.

(B) When the charge of the IF surface is neutralized by the charge on the grafted chains and the ionic strength is low, IFs experience attractive force between each other at surface separations *D*≈15– 35 *a*_0_ (6– 14 nm) due to bridging effect of grafted terminal domains. This attraction becomes weaker and turns into repulsion with increase of ionic strength as a result of electrostatic screening. The repulsion become stronger and longer ranged when simple aqueous electrolyte solution between the IF surfaces is replaced by a complex “broth” of amino acids—a coarse grained representation of NMF in 30% water. However, we can not confirm experimental observations of Jokura *et al.*[23] that neutral amino acids alone produce a similar effect. We have found that charged small species such as ions or charged amino acids are necessary components of NMF and their role is to decrease electrostatic forces between IF. The effect of salt when ions differ not only by their charge but also by size should be investigated using more complicated model for salt molecules or/and by other simulation methods.

(C) At low ionic strength the attraction between the IF surfaces can be obtained only when the charge on the surface is fully compensated by the charge on the chains and ions. That occurs only when the surface charge is equal or slightly higher than the charge of the grafted terminal domains. Therefore, we propose that: (i) negative charge of the IF helical part is equal in absolute value or slightly higher than the positive charge of the IF terminal domains; (ii) the function of NMF is to prevent the attractive forces between protruding terminal domains and IF helical cores. When NMF are removed or their amount is highly reduced these attractive forces “glue” keratin Intermediate Filaments and reduce the elasticity of the corneocytes.

## Abbreviations

NMF: Natural moisturizing factors; SC: Stratum corneum; K: Keratin; K1/K10: Keratin 1/Keratin 10; IF: Intermediate filament; SCF: Self-consistent field; AA: Amino acid model for unstructured domains; PG: Polyglycine model for unstructured domains.

## Competing interests

The authors declare competing financial interests. PBW and MGN are staff scientists whose salaries are paid by Unilever. Unilever has paid the article processing charge. PBW and MGN declare they hold Unilever shares.

## Authors’ contributions

AA and EJP performed the modelling. AA and PBW wrote the paper. MGN supervised the project and reviewed the paper. All authors read and approved the final manuscript.
